# Management of high-grade kidney trauma on bilateral polycystic kidney disease: A case report

**DOI:** 10.1016/j.ijscr.2024.110158

**Published:** 2024-08-13

**Authors:** Anastasia Pearl Angeli, Soetojo Wirjopranoto, Yufi Aulia Azmi, Antonius Galih Pranesdha Putra, Kevin Muliawan Soetanto

**Affiliations:** a Faculty of Medicine, Universitas Airlangga, Surabaya, Indonesia; b Department of Urology, Faculty of Medicine Universitas Airlangga – Dr. Soetomo General Academic Hospital, Surabaya, Indonesia; c Department of Health Sciences, University of Groningen, University Medical Center Groningen, Groningen, the Netherlands; d Department of Immunology, Faculty of Medicine Siriraj Hospital, Mahidol University, Bangkok, Thailand

**Keywords:** High-grade kidney trauma, Polycystic kidney disease, Mortality

## Abstract

**Background:**

The kidneys are the genitourinary organs most susceptible to trauma. One case is high-grade kidney trauma that can lead to kidney failure, such as Polycystic Kidney Disease (PKD). Here, we report a case of high-grade kidney trauma on PKD.

**Case report:**

A 28-year-old man was involved in a traffic accident and was diagnosed with a left kidney rupture. There was minimal free fluid in the abdominal cavum and left pleural effusion. The results of USG in the left kidney showed a rupture in the posterior part of the cortex-medulla reaching the calyx, accompanied by a left posterior peri-renal hematoma and a PKD in the right kidney. In the CT scan examination, the hematoma extended to the lower left retroperitoneum and peripancreatic. The size of the left kidney was enlarged. In the right kidney, PKD was accompanied by an enlargement of the kidney size, but no rupture was obtained. Patient had been diagnosed with high-grade kidney trauma (AAST Grade IV). The patient was given conservative therapy. He was alive and discharged from the hospital.

**Clinical discussion:**

Non-operative management (NOM) is the standard in kidney trauma management, with good outcomes in preventing morbidity and mortality. The trend toward this procedure results in a decrease in the number of unnecessary nephrectomies and a potential improvement in the quality of patient inhalation. Ultrasound and CT scan examinations are important markers.

**Conclusion:**

The management of high-grade kidney trauma on PKD can be carried out conservatively and show good patient outcomes.

## Highlights

• Non-operative management is safe and effective for hemodynamically stable patients with high-grade kidney trauma.
• Conservative management of high-grade kidney trauma on Bilateral Polycystic Kidney Disease is possible.
• Clinical and laboratory examination, important things on high-grade kidney trauma on Bilateral Kidney Disease

## 1 Introduction

The kidneys are the genitourinary organs that are most susceptible to trauma. Kidney and urogenital injuries occur in about 10–20 % of abdominal trauma in adults and children [1]. Other research stated that kidney trauma accounted for about 1 % to 5 % of all trauma patients, most of which were caused by blunt abdominal trauma (80 % to 90 %). Of the patients presented with blunt abdominal trauma, 8 % to 10 % would have a kidney injury, while 6 % would have a kidney injury if it penetrated. Blunt or penetrating traumatic kidney injury had an 86 % relationship rate with concomitant injuries [2,3].

The most common mechanism for kidney injury is blunt trauma (mainly by motor vehicle accidents and falls), while penetrating trauma (mainly caused by firearms and stab wounds) comprises the rest. Kidney trauma can cause injury to the parenchyma or blood vessels of the kidneys, which can cause bleeding or injury to the collection system with the possibility of urine leakage. Active bleeding is a complication of critical traumatic kidney injury and guides the acute management of the injury. Delayed bleeding is noted within 2 to 3 weeks of the injury and is usually caused by arteriovenous malformations (AVMs) or pseudoaneurysms [4]. The most common complications after NOM of high-grade traumatic kidney injury are hematuria, fever, acute kidney injury, and urinoma compared to wound infections, perinephric abscesses, and urinary tract infections after surgical management of these injuries [5].

Morbidity and mortality are highly dependent on the severity of injuries, concurrent injuries, and management interventions [6]. Non-operative management has become the standard in kidney trauma management, with good outcomes in morbidity and mortality [7]. A systematic review and meta-analysis by Prihadi et al. (2024) found that when they compared it to operative management, conservative management for high-grade renal trauma patients—especially those who were hemodynamically stable—carried a lower risk of death and a lower likelihood of necessitating a nephrectomy [8]. Urinary tract infections, recurrent hematuria, hypertension, urinoma, and ileus are among the complications associated with conservative therapy [9]. When conservative care for Grade IV renal damage is implemented successfully, it necessitates ongoing clinical and radiological surveillance. In stable patients, kidney salvage is possible with the proper management [10].

In general, an interprofessional approach to patient care among interprofessional healthcare professionals should be the goal of any healthcare facility. Optimal management should consider anatomical injuries, hemodynamic status, and associated injuries [1].

Patients with other comorbidities can have their condition worsen, as is the case with PKD patients. Cystic kidney disease is a common cause of end-stage kidney disease in children and adults. Autosomal dominant polycystic kidney disease (ADPKD) and autosomal recessive polycystic kidney disease (ARPKD) are cilia-related disorders and the two main forms of monogenic cystic kidney disease. ADPKD is a common disease that is mostly present in adults, while ARPKD is a rarer and often more severe form of PKD that usually appears perinatal or in early childhood [11]. There is a risk that this disease will develop into chronic kidney disease (CKD) [12,13]. This case report explores the outcome of high-grade kidney trauma on PKD. The writing of this case report follows the SCARE guidelines [14]. This case report reviews the current status and indications for conservative management of grade IV trauma with an emphasis on follow-up and complications.

## 2 Case report

**Fig. 1:**
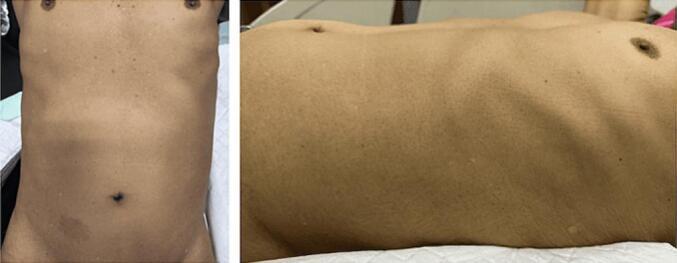
Clinical picture of the patient.

A 28-year-old man was involved in a traffic accident and was diagnosed with a left kidney rupture. In the laboratory examination, the results of the initial laboratory examination were obtained: Hb/Leu/PLT: 12.4/34.29/303. Afterwards, the laboratory examination showed Hb/Leu/PLT: 10.5/20.60/266, Ureum/Creatinin serum: 31.00/0.91, Na/K/Cl: 134/3.39/98. Urinalysis inspection showed yellow colour, slightly cloudy clarity, leu negative, nitrite negative, and erythrocytes ≥250/ul, ++++. The urine culture showed no germ growth. On a physical examination, as shown in Fig. 1, there was no bruise on the left flank.

**Fig. 2:**
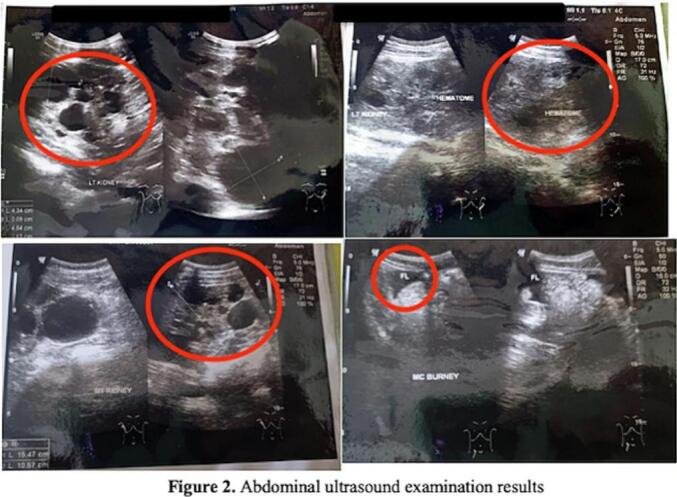
Abdominal ultrasound examination results.

In the abdominal ultrasound examination, a picture of bilateral polycystic kidney disease was obtained. On abdominal ultrasound, minimal free fluid existed in the abdominal cavum and left pleural effusion. An image of a polycystic kidney in the left kidney accompanied by a rupture in the posterior part of the cortex-medulla reached the calyx, accompanied by a peri-para hematoma of the left posterior kidney measuring 9.9 × 8.9 × 16.5 cm and polycystic kidney in the right kidney. Fig. 2 shows the ultrasound results.

**Fig. 3:**
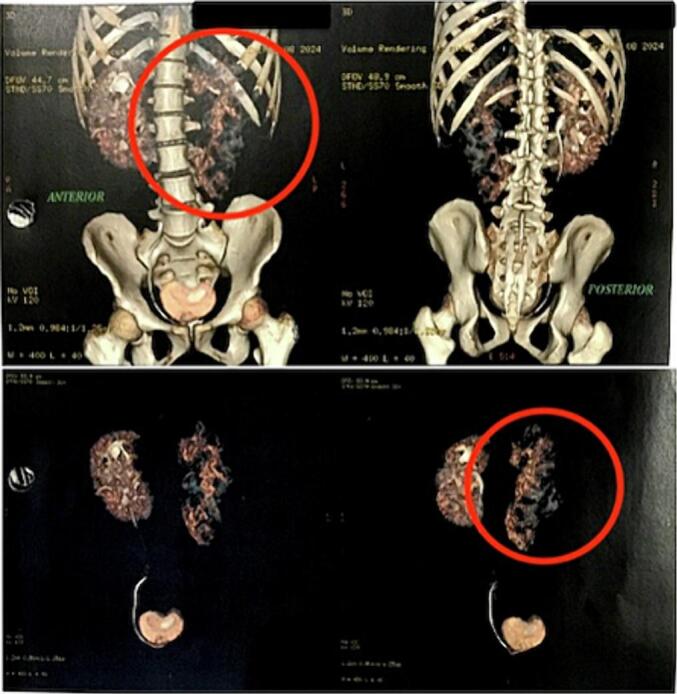
CT scan results.

In the CT-Scan examination with and without three-phase contrast, polycystic kidney was found in the left kidney accompanied by a rupture in the posterior part of the cortex-medulla reaching calyx, accompanied by a peri-para hematoma of the left posterior kidney measuring 16.7 × 10.6 × 9 cm. The hematoma extended to the lower left retroperitoneum & peripancreatic. The size of the left kidney was enlarged. The polycystic kidney was obtained in the right kidney, accompanied by an enlargement of the kidney size, but no rupture was obtained. Fig. 3 shows the results of the CT scan.

Patient with the diagnosis of high-grade kidney trauma (AAST Grade IV) was managed conservatively. This was done because the patient's hemodynamics was stable. He was placed in the Intensive Care Unit (ICU) to observe the vital signs. Creatinine levels and glomerular filtration rates were measured daily. The glomerular filtration rate (GFR) results on the first day were 100.5 mL/min/1.73 m^2^. The last condition showed the patient was alive and discharged from the hospital on day 7. When discharged from the hospital, laboratory results were Hb/Leu/PLT: 10.3/8.50/563, Creatinin serum: 0.7, GFR:134.6.9 mL/min/1.73 m^2^, Na/K: 134/3.81. After discharge from the hospital, the patient checked back at the clinic and showed that the patient was in good condition. The patient received an explanation and counselling regarding the condition of his kidney function in the future. Imaging studies were performed after admission to evaluate for further complications. Results showed the patient was in good condition. The patient did not experience any further complications, such as urinoma.

## 3 Discussion

In case, patients experience high-grade Kidney Trauma AAST Grade IV. Patients can be treated with conservative therapy. The patient's final condition is alive and can be discharged. Previous research has found that although kidney trauma is still a significant cause of morbidity, kidney loss has decreased due to the “standard of care” approach in non-operative management for hemodynamically stable patients regardless of the extent of the injury [6]. For patients with stable hemodynamics who have experienced renal trauma, non-operative care is safe and produces satisfactory results [15]. When treated with NOM, the majority of patients with blunt and penetrating renal trauma who are hemodynamically stable have a favourable prognosis. Other critical organ damage, intra- and extra-abdominal, deteriorates the patient's state of health and impacts the prognosis [16]. Urinary tract infections, recurrent hematuria, hypertension, urinoma, and ileus are among the complications associated with conservative therapy [9]. When conservative care for Grade IV renal damage is implemented successfully, it necessitates ongoing clinical and radiological surveillance. Kidney salvage is possible in stable patients with proper management [10]. The reporting results showed that the final condition of the patient was still alive and discharged from the hospital on day 7. After discharge from the hospital, the patient was checked back at the clinic and found in good condition. The patient received an explanation and counselling regarding the condition of his kidney function in the future. Imaging studies were performed after admission to evaluate for further complications. Results showed the patient was in good condition. The patient did not experience any further complications, such as urinoma. The study found urinoma formation in 1.2 % of the DJ stent group and 0.4 % with pyeloureteral stents [17]. In these cases, there was no DJ stent placement.

Pediatric kidney trauma can be successfully managed non-operatively in more than two-thirds of cases in middle-income countries. High rates of kidney injury do not predict the need for surgery or nephrectomy and can be managed non-operatively [18]. Approximately 50 % of all high-grade kidney trauma has a related collection system injury. Although most of these collection system injuries will heal spontaneously, about 20–30 % of these injuries are managed with ureteral stents [19]. Non-operative management has become the standard in kidney trauma management, with good outcomes in morbidity and mortality. This resulted in a decrease in the number of nephrectomies, unnecessary iatrogenic, and a potential increase in quality of life. When invasive treatment is required, angioembolization for active bleeding or nephrorrhaphy is usually sufficient [7]. As with all traumatic conditions, urogenital trauma management should be multidisciplinary, including urologists, interventional radiologists, and trauma surgeons, as well as emergency physicians and ICUs [1].

In these cases, ultrasound and CT scans are important markers. The modality of choice in patients with suspected kidney trauma is computed contrast-enhanced tomography with immediate and delayed imaging. A CT scan describes the findings of injury and the severity that will be seen if surgery is performed, and contrast allows evaluation of the kidney vessels. Standard laboratories must be obtained, for example, a complete blood count (CBC), a comprehensive metabolic panel (CMP), a coagulation panel, and lactate and urine for analysis. This case report also explores urine culture and laboratory [6].

In this case, the patient had an accident. Chronic kidney disease and kidney injury have traditionally been considered separate entities with different etiologies. This view has changed in recent years, with chronic kidney disease being recognized as a major risk factor for the development of new acute kidney injury, and acute kidney injury is now accepted as a de novo or accelerated cause of chronic and end-stage kidney disease. Patients with pre-existing chronic kidney disease appear to be less able to make complete ‘adaptive’ repairs after an acute injury and instead repair them maladaptively, with accelerated fibrosis and decreased rates of kidney function [20]. Patients who experience acute kidney injury after major surgery are at high risk of developing severe CKD or worsening of pre-existing CKD and other cardiovascular clinical outcomes [21]. Patients with other comorbidities can have their condition worsen, as is the case with PKD patients. Cystic kidney disease is a common cause of end-stage kidney disease in children and adults [11]. There is a risk that this disease will develop into chronic kidney disease (CKD) [12,13]. In this case, neither of these conditions leads to a poor prognosis.

## 4 Conclusion

The management of high-grade kidney trauma in polycystic kidney disease cases can be carried out conservatively and show good patient outcomes. Non-operative management (NOM) is the standard in kidney trauma management, with good outcomes in preventing morbidity and mortality. The trend toward this procedure results in a decrease in unnecessary nephrectomies and a potential improvement in the quality of patient inhalation. Ultrasound and CT scan examinations are essential markers.

## Consent

Studies on patients or volunteers require ethics committee approval and fully informed written consent Written informed consent was obtained from the patient for publication and any accompanying images. A copy of the written consent is available for review by the Editor-in-Chief of this journal on request.

## Ethical approval

Ethical approval has been acquired in this study by Health Research Ethics Committee of Dr. Soetomo General-Academic Hospital, Surabaya, Indonesia.

## Funding

This research did not receive any specific grant from funding agencies in the public, commercial, or not-for-profit sectors.

## CRediT authorship contribution statement

Anastasia Pearl Angeli: Conceptualization, Methodology, Data Curation, Investigation, Writing-Original draft preparation.

Antonius Galih Pranesdha Putra: Conceptualization, Data Curation, Writing-Original draft preparation.

Yufi Aulia Azmi: Data Curation, Writing original draft-Reviewing, and Editing.

Kevin Muliawan Soetanto: Data Curation, Writing original draft-Reviewing, and Editing.

Soetojo Wirjopranoto: Writing original draft, Reviewing, Supervision, Validation.

## Guarantor

Soetojo Wirjopranoto.

## Declaration of competing interest

The authors declare that there is no conflict of interest.

## References

[1] Coccolini F., Moore E.E., Kluger Y., Biffl W., Leppaniemi A., Matsumura Y. (2019). Kidney and uro-trauma: WSES-AAST guidelines. World J. Emerg. Surg..

[2] Voelzke B.B., Leddy L. (2014). The epidemiology of renal trauma. Transl. Androl. Urol..

[3] Heller M.T., Schnor N. (2014). MDCT of renal trauma: correlation to AAST organ injury scale. Clin. Imaging [Internet]..

[4] Starnes M., Demetriades D., Hadjizacharia P., Inaba K., Best C., Chan L. (2010 Apr 1). Complications following renal trauma. Arch. Surg. [Internet]..

[5] Morey A.F., Broghammer J.A., Hollowell C.M., McKibben M.J.S.L. (2021). Urotrauma guideline 2020: AUA guideline. J. Urol..

[6] Singh S.S.K. (2024). https://www.ncbi.nlm.nih.gov/books/NBK532896/%0A.

[7] Petrone P., Perez-Calvo J., Brathwaite C.E.M., Islam S., Joseph D.K. (2020). Traumatic kidney injuries: a systematic review and meta-analysis. Int. J. Surg. [Internet]..

[8] Prihadi J., Hengky A., Lionardi S. (2024). Conservative management in high-grade renal trauma: a systematic review and meta-analysis. BJU Int..

[9] Ponnusamy P., Poovathai S., Ramakrishnan Rajkumar, Sangreshi V. (2023). Conservative management a safer option in high-grade renal injuries: our institutional experience. Asian. J. Med. Sci..

[10] Prakash S.V., Mohan C.G., Reddy V.B., Reddy V.K., Kumar A.R.U. (2015). Salvageability of kidney in grade IV renal trauma by minimally invasive treatment methods. J. Emerg. Trauma Shock.

[11] Igarashi P., Somlo S. (2007). Polycystic kidney disease. J. Am. Soc. Nephrol. [Internet]..

[12] Iliuta I.A., Win A.Z., Lanktree M.B., Lee S.H., Pourafkari M., Nasri F. (2023). Atypical polycystic kidney disease as defined by imaging. Sci. Rep. [Internet]..

[13] Chebib F.T., Torres V.E. (2021). Assessing risk of rapid progression in autosomal dominant polycystic kidney disease and special considerations for disease-modifying therapy. Am. J. Kidney Dis. [Internet]..

[14] Sohrabi C., Mathew G., Maria N., Kerwan A., Franchi T., Agha T. (2023). The SCARE 2023 guideline: updating consensus Surgical CAse REport (SCARE) guidelines. Int. J. Surg. L. Engl..

[15] Palinrungi M.A., Faruk M., Christeven R. (2023). Traumatic kidney injury: a 6-year retrospective study in childhood and adolescence. Res. Reports Urol..

[16] Syarif Palinrungi A.M., Kholis K., Palinrungi M.A., Syahrir S., Sunggiardi R. (2020 Nov). Renal trauma: a 5-year retrospective review in single institution. African. J. Urol..

[17] Garg R.K., Menon P., Narasimha Rao K.L., Arora S.B.Y. (2015). Pyeloplasty for hydronephrosis: issues of double J stent versus nephrostomy tube as drainage technique. J. Indian Assoc. Pediatr. Surg..

[18] Thirayan V., Kong V.Y., Elsabagh A., Xu W., Rajaretnam N., Conradie B. (2023). High-grade renal trauma in children and adolescents can be successfully managed non-operatively. S. Afr. J. Surg..

[19] Locke J.A., Neu S., Navaratnam R., Phillips A., Nathens A.B., Herschorn S., Kodama R. (2021). Management of high-grade renal traumas with collecting system injuries. Can. Urol. Assoc. J..

[20] Ferenbach D.A., Bonventre J.V., Division B.E., Hospital W. (2017). Laboratory to the clinic. Nephrol. Ther..

[21] Ohlmeier C., Schuchhardt J., Bauer C., Brinker M., Kong S.X., Scott C. (2023 Dec 1). Risk of chronic kidney disease in patients with acute kidney injury following a major surgery: a US claims database analysis. Clin Kidney J [Internet]..

